# 4-Butyl-3-(3,5-dimethoxy­phen­yl)-4-meth­oxy-2-(triisopropyl­silyl)cyclopent-2-enone

**DOI:** 10.1107/S1600536808002055

**Published:** 2008-01-25

**Authors:** Zhenyu Zhao, Yunhui Zhang, Xinghua Jin, Xinjian Yang

**Affiliations:** a School of Pharmaceutical Science and Technology, Tianjin University, Tianjin 300072, People’s Republic of China; b Changzheng Hospital, Tianjin 300120, People’s Republic of China

## Abstract

The title mol­ecule, C_27_H_44_O_4_Si, bears a bulky triisopropyl­silyl group. The cyclopentene ring adopts an envelope conformation; the plane of its four coplanar C atoms and the benzene ring make a dihedral angle of 73.2 (6)°.

## Related literature

For related literature, see: Allen *et al.* (1987 ▶); Frontier & Collison (2005 ▶); Geis & Schmalz (1998 ▶); Roberts *et al.* (2002 ▶); Shi *et al.* (2005 ▶); Tanaka & Fu (2001 ▶); Li *et al.* (2007 ▶, 2008 ▶).
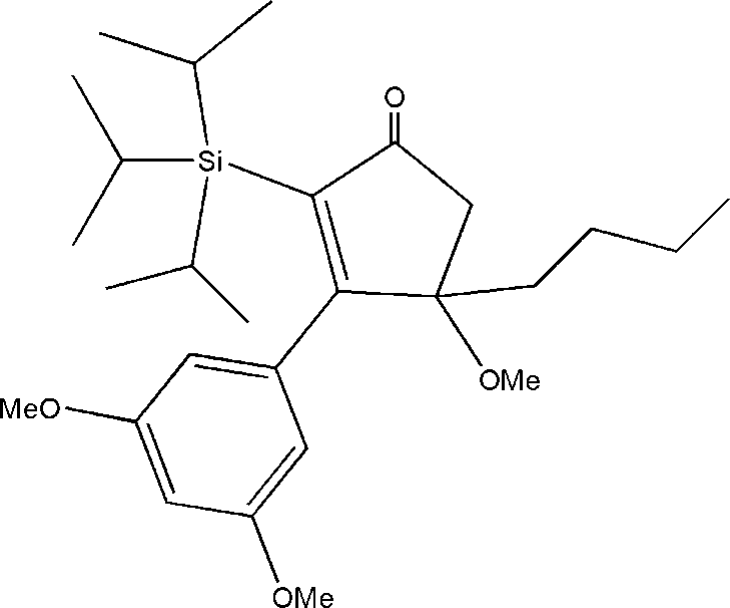


## Experimental

### 

#### Crystal data

C_27_H_44_O_4_Si
*M* *_r_* = 460.71
Monoclinic, 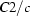

*a* = 39.294 (4) Å
*b* = 8.3327 (10) Å
*c* = 29.274 (3) Å
β = 146.207 (8)°
*V* = 5331.1 (15) Å^3^
*Z* = 8
Mo *K*α radiation
μ = 0.12 mm^−1^
*T* = 113 (2) K
0.26 × 0.24 × 0.10 mm

#### Data collection

Rigaku Saturn diffractometer
Absorption correction: multi-scan (*CrystalClear*; Rigaku, 2005) *T*_min_ = 0.961, *T*_max_ = 0.988
32688 measured reflections
6356 independent reflections
5271 reflections with *I* > 2σ(*I*)
*R*_int_ = 0.051

#### Refinement

*R*[*F* ^2^ > 2σ(*F* ^2^)] = 0.049
*wR*(*F* ^2^) = 0.123
*S* = 1.09
6356 reflections
291 parameters
H-atom parameters constrained
Δρ_max_ = 0.29 e Å^−3^
Δρ_min_ = −0.34 e Å^−3^

### 

Data collection: *CrystalClear* (Rigaku, 2005 ▶); cell refinement: *CrystalClear*; data reduction: *CrystalClear*; program(s) used to solve structure: *SHELXS97* (Sheldrick, 2008 ▶); program(s) used to refine structure: *SHELXL97* (Sheldrick, 2008 ▶); molecular graphics: *SHELXTL* (Sheldrick, 2008); software used to prepare material for publication: *CrystalStructure* (Rigaku, 2005).

## Supplementary Material

Crystal structure: contains datablocks global, I. DOI: 10.1107/S1600536808002055/rk2071sup1.cif

Structure factors: contains datablocks I. DOI: 10.1107/S1600536808002055/rk2071Isup2.hkl

Additional supplementary materials:  crystallographic information; 3D view; checkCIF report

## Figures and Tables

**Fig. 1.**
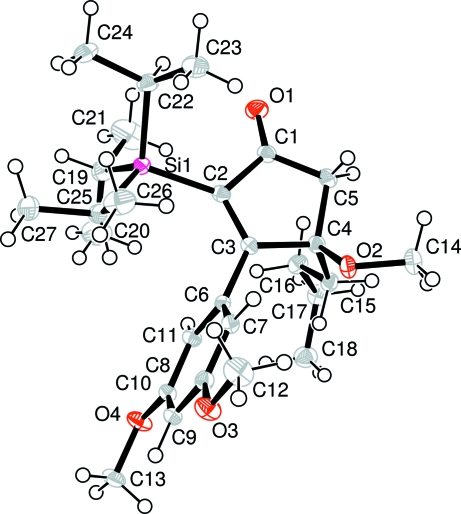
The molecule structure of title compound with atom numbering scheme. Displacement ellipsoids are drawn at the 50% probability level. H atoms are presented as a small spheres with the arbitrary radius.

## supplementary crystallographic information

### Comment

Cyclopentenones are common structural units in bioactive natural products and useful building blocks. For a review see: (Roberts *et al.*, 2002). While various approaches such as the Nazarov reaction (Frontier & Collison, 2005), Pauson–Khand reaction (Geis & Schmalz, 1998), Rautenstrauch rearrangement reaction (Shi *et al.*, 2005), and intramolecular hydroacylation reaction (Tanaka & Fu, 2001) have been developed for construction of this important ring system. The title molecule was synthesized by [4 + 1] reaction of triisopropylsilyl vinyl ketene with Kobrich reagent (Li *et al.*, 2007; Li *et al.*, 2008).

The molecule structure of C_27_H_44_O_4_Si, is illustrated on Fig.1. The molecule consists of one cyclopentenone (C1–C5) and one phenyl ring (C6–C11). These rings are form the dihedral angle 73.2 (6)°. In the title molecule all bond lengths and bond angles are agree with published (Allen *et al.*, 1987).

### Experimental

A solution of the tricarbonyl chromium complexed silyl vinylketene (0.5 mmol) and CH_2_I_2_ or Br_2_CHCH_3_ (0.75 mmol) in 5 ml of *Et*_2_O was cooled to 195 K. The *n*–*Bu*Li (0.75 mmol) was added within 10 min by syringe. After stirring at 195 K for 1 h, the solution was allowed to warm to room temperature and stirred overnight. The resulting solution was diluted with 30 ml of *Et*_2_O and extracted with saturated NaCl (3 × 10 ml). The aqueous layer was backextracted with *Et*_2_O (3 × 10 ml). The combined organic layer was dried over anhydrous Na_2_SO_4_. After filtration, the solvent was removed under reduced pressure and the residue was purified *via* flash chromatography (SiO_2_) to afford the cyclopentenone compound (yield 78%, m.p. 332–333 K). ^1^H NMR (CDCl_3_): 6.46 (t, 1H), 6.36 (d, 2H), 3.79 (s, 6H), 3.24 (s, 3H), 2.66 (d, 1H), 2.42 (d, 1H), 1.68–1.63 (m, 1H), 1.44–1.39 (m, 1H), 1.35–1.12 (m, 7H), 0.98 (d, 9H), 0.94 (d, 9H), 0.84 (t, 3H) p.p.m.; ^13^C NMR (CDCl_3_): 210.0, 183.3, 159.8, 143.0, 137.5, 106.2, 100.2, 87.0, 55.3, 50.7, 42.2, 36.9, 26.5, 23.0, 19.2, 19.1, 14.0, 11.4 p.p.m..

### Refinement

All H atoms were placed in calculated positions and refined as riding, with C—H = 0.95–1.00Å and with *U*_iso_(H) = 1.2 *U*_eq_(C).

### Crystal data

**Table:**

C_27_H_44_O_4_Si	*F*_000_ = 2016
*M**_r_* = 460.71	*D*_x_ = 1.148 Mg m^−^^3^
Monoclinic, *C*2/*c*	Melting point = 332–333 K
Hall symbol: -C 2yc	Mo *K*α radiation λ = 0.71070 Å
*a* = 39.294 (4) Å	Cell parameters from 374 reflections
*b* = 8.3327 (10) Å	θ = 1.9–27.9º
*c* = 29.274 (3) Å	µ = 0.12 mm^−^^1^
β = 146.207 (8)º	*T* = 113 (2) K
*V* = 5331.1 (15) Å^3^	Block, colourless
*Z* = 8	0.26 × 0.24 × 0.10 mm

### Data collection

**Table:**

Rigaku Saturn diffractometer	6356 independent reflections
Radiation source: rotating anode	5271 reflections with *I* > 2σ(*I*)
Monochromator: confocal	*R*_int_ = 0.051
Detector resolution: 7.31 pixels mm^-1^	θ_max_ = 27.9º
*T* = 113(2) K	θ_min_ = 1.9º
ω scans	*h* = −51→49
Absorption correction: multi-scan(CrystalClear; Rigaku, 2005)	*k* = −10→10
*T*_min_ = 0.961, *T*_max_ = 0.988	*l* = −37→38
32688 measured reflections	

### Refinement

**Table:**

Refinement on *F*^2^	Hydrogen site location: inferred from neighbouring sites
Least-squares matrix: full	H-atom parameters constrained
*R*[*F*^2^ > 2σ(*F*^2^)] = 0.049	*w* = 1/[σ^2^(*F*_o_^2^) + (0.0613*P*)^2^ + 2.2212*P*] where *P* = (*F*_o_^2^ + 2*F*_c_^2^)/3
*wR*(*F*^2^) = 0.123	(Δ/σ)_max_ = 0.001
*S* = 1.09	Δρ_max_ = 0.29 e Å^−^^3^
6356 reflections	Δρ_min_ = −0.34 e Å^−^^3^
291 parameters	Extinction correction: SHELXL97 (Sheldrick, 1997), Fc^*^=kFc[1+0.001xFc^2^λ^3^/sin(2θ)]^-1/4^
Primary atom site location: structure-invariant direct methods	Extinction coefficient: 0.0029 (3)
Secondary atom site location: difference Fourier map	

### Special details

**Table:**

Geometry. All s.u.'s (except the s.u.'s in the dihedral angle between two l.s. planes) are estimated using the full covariance matrix. The cell s.u.'s are taken into account individually in the estimation of s.u.'s in distances, angles and torsion angles; correlations between s.u.'s in cell parameters are only used when they are defined by crystal symmetry. An approximate (isotropic) treatment of cell s.u.'s is used for estimating s.u.'s involving l.s. planes.
Refinement. Refinement of *F*^2^ against ALL reflections. The weighted *R*–factor *wR* and goodness of fit *S* are based on *F*^2^, conventional *R*–factors *R* are based on *F*, with *F* set to zero for negative *F*^2^. The threshold expression of *F*^2^ > 2σ(*F*^2^) is used only for calculating *R*–factors(gt) *etc*. and is not relevant to the choice of reflections for refinement. *R*–factors based on *F*^2^ are statistically about twice as large as those based on *F*, and *R*–factors based on ALL data will be even larger.

### Fractional atomic coordinates and isotropic or equivalent isotropic displacement parameters (Å^2^)

**Table:**

	*x*	*y*	*z*	*U*_iso_*/*U*_eq_	
Si1	0.060076 (17)	0.46916 (5)	0.45150 (2)	0.01708 (12)	
O1	0.14533 (5)	0.38982 (14)	0.63443 (6)	0.0260 (3)	
O2	0.22955 (5)	0.04752 (12)	0.61756 (6)	0.0191 (2)	
O3	0.13563 (5)	0.06140 (13)	0.35883 (7)	0.0249 (3)	
O4	0.21072 (5)	0.59302 (13)	0.45866 (7)	0.0257 (3)	
C1	0.16047 (6)	0.32410 (18)	0.61599 (8)	0.0185 (3)	
C2	0.13558 (6)	0.35913 (17)	0.54188 (8)	0.0162 (3)	
C3	0.17437 (6)	0.29104 (16)	0.55487 (8)	0.0148 (3)	
C4	0.22794 (6)	0.20168 (17)	0.63719 (8)	0.0166 (3)	
C5	0.20858 (6)	0.19464 (18)	0.66571 (8)	0.0198 (3)	
H5A	0.1914	0.0881	0.6542	0.024*	
H5B	0.2437	0.2170	0.7253	0.024*	
C6	0.17257 (6)	0.30081 (16)	0.50223 (8)	0.0153 (3)	
C7	0.15364 (6)	0.16823 (17)	0.45500 (8)	0.0171 (3)	
H7	0.1411	0.0716	0.4555	0.021*	
C8	0.15356 (6)	0.18095 (17)	0.40790 (8)	0.0184 (3)	
C9	0.17267 (6)	0.32121 (18)	0.40742 (8)	0.0192 (3)	
H9	0.1727	0.3280	0.3751	0.023*	
C10	0.19146 (6)	0.44980 (17)	0.45446 (9)	0.0179 (3)	
C11	0.19134 (6)	0.44033 (17)	0.50188 (8)	0.0179 (3)	
H11	0.2042	0.5297	0.5339	0.021*	
C12	0.11224 (8)	−0.08178 (19)	0.35246 (11)	0.0313 (4)	
H12A	0.1013	−0.1574	0.3159	0.038*	
H12B	0.0758	−0.0566	0.3300	0.038*	
H12C	0.1435	−0.1301	0.4070	0.038*	
C13	0.21186 (7)	0.6089 (2)	0.41147 (10)	0.0250 (3)	
H13A	0.2264	0.7164	0.4195	0.030*	
H13B	0.1705	0.5931	0.3532	0.030*	
H13C	0.2394	0.5282	0.4292	0.030*	
C14	0.27015 (7)	−0.06577 (18)	0.68287 (9)	0.0252 (3)	
H14A	0.2682	−0.1668	0.6636	0.030*	
H14B	0.2582	−0.0848	0.7014	0.030*	
H14C	0.3120	−0.0237	0.7286	0.030*	
C15	0.28884 (6)	0.29106 (17)	0.69819 (8)	0.0180 (3)	
H15A	0.3219	0.2273	0.7494	0.022*	
H15B	0.2975	0.2968	0.6746	0.022*	
C16	0.29095 (7)	0.46110 (18)	0.72010 (9)	0.0214 (3)	
H16A	0.2767	0.4582	0.7364	0.026*	
H16B	0.2623	0.5297	0.6707	0.026*	
C17	0.35466 (7)	0.53686 (19)	0.78964 (9)	0.0233 (3)	
H17A	0.3534	0.6409	0.8046	0.028*	
H17B	0.3837	0.4662	0.8384	0.028*	
C18	0.37830 (8)	0.5645 (2)	0.76797 (11)	0.0303 (4)	
H18A	0.4193	0.6120	0.8151	0.036*	
H18B	0.3506	0.6374	0.7209	0.036*	
H18C	0.3803	0.4618	0.7539	0.036*	
C19	0.07163 (7)	0.69311 (19)	0.47085 (10)	0.0267 (3)	
H19	0.0304	0.7438	0.4214	0.032*	
C20	0.11217 (9)	0.7634 (2)	0.47859 (13)	0.0409 (5)	
H20A	0.0967	0.7301	0.4313	0.049*	
H20B	0.1542	0.7244	0.5293	0.049*	
H20C	0.1117	0.8808	0.4801	0.049*	
C21	0.09602 (12)	0.7453 (2)	0.54452 (14)	0.0511 (6)	
H21A	0.0701	0.7004	0.5398	0.061*	
H21B	0.0956	0.8627	0.5460	0.061*	
H21C	0.1380	0.7064	0.5951	0.061*	
C22	0.01096 (6)	0.39689 (19)	0.44564 (9)	0.0225 (3)	
H22	0.0294	0.4441	0.4944	0.027*	
C23	0.01070 (8)	0.2140 (2)	0.45283 (11)	0.0313 (4)	
H23A	0.0529	0.1748	0.5017	0.038*	
H23B	−0.0103	0.1617	0.4038	0.038*	
H23C	−0.0107	0.1890	0.4576	0.038*	
C24	−0.05484 (7)	0.4611 (2)	0.37011 (10)	0.0308 (4)	
H24A	−0.0540	0.5777	0.3666	0.037*	
H24B	−0.0760	0.4359	0.3751	0.037*	
H24C	−0.0765	0.4107	0.3204	0.037*	
C25	0.02599 (7)	0.4266 (2)	0.35538 (9)	0.0249 (3)	
H25	0.0600	0.4375	0.3707	0.030*	
C26	0.00158 (8)	0.2545 (2)	0.32357 (10)	0.0353 (4)	
H26A	0.0334	0.1784	0.3684	0.042*	
H26B	−0.0097	0.2341	0.2791	0.042*	
H26C	−0.0345	0.2413	0.3029	0.042*	
C27	−0.02368 (8)	0.5469 (3)	0.28563 (10)	0.0382 (5)	
H27A	−0.0078	0.6564	0.3063	0.046*	
H27B	−0.0596	0.5339	0.2653	0.046*	
H27C	−0.0353	0.5271	0.2408	0.046*	

### Atomic displacement parameters (Å^2^)

**Table:**

	*U*^11^	*U*^22^	*U*^33^	*U*^12^	*U*^13^	*U*^23^
Si1	0.01359 (19)	0.0209 (2)	0.01541 (19)	0.00036 (15)	0.01180 (17)	0.00101 (15)
O1	0.0252 (6)	0.0377 (7)	0.0214 (5)	0.0034 (5)	0.0206 (5)	0.0003 (5)
O2	0.0233 (5)	0.0154 (5)	0.0166 (5)	0.0039 (4)	0.0162 (5)	0.0030 (4)
O3	0.0357 (6)	0.0215 (6)	0.0267 (6)	−0.0029 (5)	0.0277 (5)	−0.0047 (4)
O4	0.0393 (6)	0.0213 (6)	0.0349 (6)	−0.0050 (5)	0.0342 (6)	−0.0016 (5)
C1	0.0163 (6)	0.0234 (7)	0.0148 (6)	−0.0019 (6)	0.0127 (6)	−0.0005 (5)
C2	0.0156 (6)	0.0189 (7)	0.0144 (6)	−0.0024 (5)	0.0125 (6)	−0.0018 (5)
C3	0.0156 (6)	0.0145 (7)	0.0134 (6)	−0.0025 (5)	0.0119 (6)	−0.0019 (5)
C4	0.0179 (7)	0.0164 (7)	0.0151 (6)	0.0014 (5)	0.0137 (6)	0.0007 (5)
C5	0.0198 (7)	0.0246 (8)	0.0158 (7)	0.0015 (6)	0.0149 (6)	0.0026 (6)
C6	0.0129 (6)	0.0188 (7)	0.0129 (6)	0.0031 (5)	0.0105 (6)	0.0027 (5)
C7	0.0175 (6)	0.0166 (7)	0.0166 (6)	0.0015 (5)	0.0140 (6)	0.0022 (5)
C8	0.0191 (7)	0.0181 (7)	0.0169 (6)	0.0025 (5)	0.0148 (6)	0.0000 (5)
C9	0.0208 (7)	0.0236 (8)	0.0174 (7)	0.0043 (6)	0.0167 (6)	0.0039 (6)
C10	0.0184 (7)	0.0186 (7)	0.0188 (7)	0.0017 (5)	0.0158 (6)	0.0034 (5)
C11	0.0190 (7)	0.0195 (7)	0.0170 (7)	−0.0011 (5)	0.0153 (6)	−0.0010 (5)
C12	0.0424 (10)	0.0223 (8)	0.0343 (9)	−0.0074 (7)	0.0328 (9)	−0.0087 (7)
C13	0.0290 (8)	0.0302 (9)	0.0269 (8)	−0.0033 (7)	0.0253 (7)	0.0012 (6)
C14	0.0263 (8)	0.0210 (8)	0.0208 (7)	0.0051 (6)	0.0182 (7)	0.0056 (6)
C15	0.0157 (6)	0.0201 (7)	0.0152 (6)	0.0019 (5)	0.0123 (6)	0.0005 (5)
C16	0.0206 (7)	0.0216 (8)	0.0209 (7)	−0.0004 (6)	0.0171 (7)	−0.0030 (6)
C17	0.0223 (7)	0.0230 (8)	0.0200 (7)	−0.0025 (6)	0.0167 (7)	−0.0038 (6)
C18	0.0303 (9)	0.0297 (9)	0.0350 (9)	−0.0028 (7)	0.0279 (8)	−0.0012 (7)
C19	0.0281 (8)	0.0224 (8)	0.0327 (8)	0.0022 (6)	0.0258 (8)	0.0034 (6)
C20	0.0494 (11)	0.0273 (10)	0.0606 (13)	−0.0089 (8)	0.0484 (11)	−0.0047 (9)
C21	0.0878 (17)	0.0259 (10)	0.0725 (15)	−0.0118 (10)	0.0728 (15)	−0.0132 (10)
C22	0.0166 (7)	0.0304 (9)	0.0206 (7)	−0.0015 (6)	0.0155 (6)	−0.0009 (6)
C23	0.0285 (8)	0.0318 (9)	0.0371 (9)	−0.0053 (7)	0.0279 (8)	−0.0001 (7)
C24	0.0193 (7)	0.0450 (10)	0.0288 (8)	0.0033 (7)	0.0201 (7)	0.0044 (7)
C25	0.0162 (7)	0.0404 (10)	0.0163 (7)	0.0005 (6)	0.0131 (6)	0.0019 (6)
C26	0.0277 (9)	0.0515 (11)	0.0261 (8)	−0.0100 (8)	0.0222 (8)	−0.0146 (8)
C27	0.0228 (8)	0.0643 (13)	0.0210 (8)	0.0104 (8)	0.0170 (8)	0.0124 (8)

### Geometric parameters (Å, °)

**Table:**

Si1—C22	1.8886 (15)	C15—H15A	0.9900
Si1—C25	1.8902 (16)	C15—H15B	0.9900
Si1—C19	1.8926 (17)	C16—C17	1.529 (2)
Si1—C2	1.8962 (15)	C16—H16A	0.9900
O1—C1	1.2161 (17)	C16—H16B	0.9900
O2—C14	1.4220 (17)	C17—C18	1.516 (5)
O2—C4	1.4299 (17)	C17—H17A	0.9900
O3—C8	1.3674 (17)	C17—H17B	0.9900
O3—C12	1.4206 (19)	C18—H18A	0.9800
O4—C10	1.3630 (17)	C18—H18B	0.9800
O4—C13	1.425 (4)	C18—H18C	0.9800
C1—C2	1.4905 (19)	C19—C21	1.525 (2)
C1—C5	1.511 (2)	C19—C20	1.527 (6)
C2—C3	1.351 (4)	C19—H19	1.0000
C3—C6	1.485 (4)	C20—H20A	0.9800
C3—C4	1.5366 (19)	C20—H20B	0.9800
C4—C5	1.528 (4)	C20—H20C	0.9800
C4—C15	1.5384 (19)	C21—H21A	0.9800
C5—H5A	0.9900	C21—H21B	0.9800
C5—H5B	0.9900	C21—H21C	0.9800
C6—C11	1.381 (2)	C22—C24	1.536 (2)
C6—C7	1.4057 (19)	C22—C23	1.540 (2)
C7—C8	1.380 (2)	C22—H22	1.0000
C7—H7	0.9500	C23—H23A	0.9800
C8—C9	1.396 (2)	C23—H23B	0.9800
C9—C10	1.378 (2)	C23—H23C	0.9800
C9—H9	0.9500	C24—H24A	0.9800
C10—C11	1.394 (2)	C24—H24B	0.9800
C11—H11	0.9500	C24—H24C	0.9800
C12—H12A	0.9800	C25—C26	1.536 (2)
C12—H12B	0.9800	C25—C27	1.536 (2)
C12—H12C	0.9800	C25—H25	1.0000
C13—H13A	0.9800	C26—H26A	0.9800
C13—H13B	0.9800	C26—H26B	0.9800
C13—H13C	0.9800	C26—H26C	0.9800
C14—H14A	0.9800	C27—H27A	0.9800
C14—H14B	0.9800	C27—H27B	0.9800
C14—H14C	0.9800	C27—H27C	0.9800
C15—C16	1.529 (2)		
			
C22—Si1—C25	113.49 (7)	C15—C16—C17	113.52 (12)
C22—Si1—C19	108.33 (7)	C15—C16—H16A	108.9
C25—Si1—C19	109.11 (7)	C17—C16—H16A	108.9
C22—Si1—C2	105.57 (6)	C15—C16—H16B	108.9
C25—Si1—C2	109.74 (7)	C17—C16—H16B	108.9
C19—Si1—C2	110.56 (7)	H16A—C16—H16B	107.7
C14—O2—C4	115.53 (11)	C18—C17—C16	113.59 (13)
C8—O3—C12	117.52 (12)	C18—C17—H17A	108.8
C10—O4—C13	117.17 (12)	C16—C17—H17A	108.8
O1—C1—C2	125.91 (13)	C18—C17—H17B	108.8
O1—C1—C5	125.15 (13)	C16—C17—H17B	108.8
C2—C1—C5	108.93 (12)	H17A—C17—H17B	107.7
C3—C2—C1	106.60 (12)	C17—C18—H18A	109.5
C3—C2—Si1	133.35 (11)	C17—C18—H18B	109.5
C1—C2—Si1	119.92 (10)	H18A—C18—H18B	109.5
C2—C3—C6	127.95 (12)	C17—C18—H18C	109.5
C2—C3—C4	113.55 (12)	H18A—C18—H18C	109.5
C6—C3—C4	118.40 (11)	H18B—C18—H18C	109.5
O2—C4—C5	113.17 (11)	C21—C19—C20	109.20 (16)
O2—C4—C3	105.40 (10)	C21—C19—Si1	114.66 (12)
C5—C4—C3	102.71 (11)	C20—C19—Si1	112.57 (12)
O2—C4—C15	110.85 (11)	C21—C19—H19	106.6
C5—C4—C15	112.62 (11)	C20—C19—H19	106.6
C3—C4—C15	111.59 (11)	Si1—C19—H19	106.6
C1—C5—C4	103.81 (11)	C19—C20—H20A	109.5
C1—C5—H5A	111.0	C19—C20—H20B	109.5
C4—C5—H5A	111.0	H20A—C20—H20B	109.5
C1—C5—H5B	111.0	C19—C20—H20C	109.5
C4—C5—H5B	111.0	H20A—C20—H20C	109.5
H5A—C5—H5B	109.0	H20B—C20—H20C	109.5
C11—C6—C7	120.24 (13)	C19—C21—H21A	109.5
C11—C6—C3	119.24 (12)	C19—C21—H21B	109.5
C7—C6—C3	120.49 (12)	H21A—C21—H21B	109.5
C8—C7—C6	118.83 (13)	C19—C21—H21C	109.5
C8—C7—H7	120.6	H21A—C21—H21C	109.5
C6—C7—H7	120.6	H21B—C21—H21C	109.5
O3—C8—C7	124.39 (13)	C24—C22—C23	110.32 (13)
O3—C8—C9	114.29 (12)	C24—C22—Si1	112.98 (11)
C7—C8—C9	121.32 (13)	C23—C22—Si1	115.18 (11)
C10—C9—C8	119.15 (13)	C24—C22—H22	105.9
C10—C9—H9	120.4	C23—C22—H22	105.9
C8—C9—H9	120.4	Si1—C22—H22	105.9
O4—C10—C9	124.54 (13)	C22—C23—H23A	109.5
O4—C10—C11	114.90 (13)	C22—C23—H23B	109.5
C9—C10—C11	120.56 (13)	H23A—C23—H23B	109.5
C6—C11—C10	119.91 (13)	C22—C23—H23C	109.5
C6—C11—H11	120.0	H23A—C23—H23C	109.5
C10—C11—H11	120.0	H23B—C23—H23C	109.5
O3—C12—H12A	109.5	C22—C24—H24A	109.5
O3—C12—H12B	109.5	C22—C24—H24B	109.5
H12A—C12—H12B	109.5	H24A—C24—H24B	109.5
O3—C12—H12C	109.5	C22—C24—H24C	109.5
H12A—C12—H12C	109.5	H24A—C24—H24C	109.5
H12B—C12—H12C	109.5	H24B—C24—H24C	109.5
O4—C13—H13A	109.5	C26—C25—C27	109.87 (14)
O4—C13—H13B	109.5	C26—C25—Si1	113.00 (11)
H13A—C13—H13B	109.5	C27—C25—Si1	113.70 (12)
O4—C13—H13C	109.5	C26—C25—H25	106.6
H13A—C13—H13C	109.5	C27—C25—H25	106.6
H13B—C13—H13C	109.5	Si1—C25—H25	106.6
O2—C14—H14A	109.5	C25—C26—H26A	109.5
O2—C14—H14B	109.5	C25—C26—H26B	109.5
H14A—C14—H14B	109.5	H26A—C26—H26B	109.5
O2—C14—H14C	109.5	C25—C26—H26C	109.5
H14A—C14—H14C	109.5	H26A—C26—H26C	109.5
H14B—C14—H14C	109.5	H26B—C26—H26C	109.5
C16—C15—C4	115.18 (12)	C25—C27—H27A	109.5
C16—C15—H15A	108.5	C25—C27—H27B	109.5
C4—C15—H15A	108.5	H27A—C27—H27B	109.5
C16—C15—H15B	108.5	C25—C27—H27C	109.5
C4—C15—H15B	108.5	H27A—C27—H27C	109.5
H15A—C15—H15B	107.5	H27B—C27—H27C	109.5
			
O1—C1—C2—C3	165.05 (14)	C6—C7—C8—O3	−179.32 (13)
C5—C1—C2—C3	−14.12 (16)	C6—C7—C8—C9	1.0 (2)
O1—C1—C2—Si1	−18.4 (2)	O3—C8—C9—C10	179.69 (12)
C5—C1—C2—Si1	162.46 (10)	C7—C8—C9—C10	−0.6 (2)
C22—Si1—C2—C3	139.93 (15)	C13—O4—C10—C9	−0.6 (2)
C25—Si1—C2—C3	17.28 (17)	C13—O4—C10—C11	179.85 (13)
C19—Si1—C2—C3	−103.13 (15)	C8—C9—C10—O4	−179.59 (13)
C22—Si1—C2—C1	−35.56 (13)	C8—C9—C10—C11	−0.1 (2)
C25—Si1—C2—C1	−158.21 (11)	C7—C6—C11—C10	0.1 (2)
C19—Si1—C2—C1	81.38 (12)	C3—C6—C11—C10	178.29 (13)
C1—C2—C3—C6	−175.35 (13)	O4—C10—C11—C6	179.88 (12)
Si1—C2—C3—C6	8.7 (2)	C9—C10—C11—C6	0.3 (2)
C1—C2—C3—C4	1.09 (16)	O2—C4—C15—C16	177.07 (11)
Si1—C2—C3—C4	−174.83 (11)	C5—C4—C15—C16	−55.00 (16)
C14—O2—C4—C5	−59.75 (15)	C3—C4—C15—C16	59.92 (15)
C14—O2—C4—C3	−171.23 (11)	C4—C15—C16—C17	171.84 (12)
C14—O2—C4—C15	67.88 (15)	C15—C16—C17—C18	64.92 (17)
C2—C3—C4—O2	130.51 (12)	C22—Si1—C19—C21	53.07 (15)
C6—C3—C4—O2	−52.67 (15)	C25—Si1—C19—C21	177.07 (14)
C2—C3—C4—C5	11.79 (15)	C2—Si1—C19—C21	−62.15 (15)
C6—C3—C4—C5	−171.39 (12)	C22—Si1—C19—C20	178.71 (12)
C2—C3—C4—C15	−109.09 (14)	C25—Si1—C19—C20	−57.29 (14)
C6—C3—C4—C15	67.72 (15)	C2—Si1—C19—C20	63.48 (14)
O1—C1—C5—C4	−158.20 (14)	C25—Si1—C22—C24	−54.04 (14)
C2—C1—C5—C4	20.98 (15)	C19—Si1—C22—C24	67.30 (13)
O2—C4—C5—C1	−132.07 (12)	C2—Si1—C22—C24	−174.26 (11)
C3—C4—C5—C1	−18.94 (14)	C25—Si1—C22—C23	73.98 (13)
C15—C4—C5—C1	101.23 (13)	C19—Si1—C22—C23	−164.68 (12)
C2—C3—C6—C11	75.56 (19)	C2—Si1—C22—C23	−46.23 (13)
C4—C3—C6—C11	−100.74 (15)	C22—Si1—C25—C26	−46.17 (13)
C2—C3—C6—C7	−106.22 (17)	C19—Si1—C25—C26	−167.07 (11)
C4—C3—C6—C7	77.48 (16)	C2—Si1—C25—C26	71.66 (12)
C11—C6—C7—C8	−0.7 (2)	C22—Si1—C25—C27	79.96 (13)
C3—C6—C7—C8	−178.91 (12)	C19—Si1—C25—C27	−40.94 (14)
C12—O3—C8—C7	3.8 (2)	C2—Si1—C25—C27	−162.22 (11)
C12—O3—C8—C9	−176.46 (13)		

## References

[bb1] Allen, F. H., Kennard, O., Watson, D., Brammer, L., Orpen, A. G. & Taylor, R. (1987). *J. Chem. Soc. Perkin Trans. 2*, pp. S1–S19.

[bb2] Frontier, A. J. & Collison, C. (2005). *Tetrahedron*, **61**, 7577–7606.

[bb3] Geis, O. & Schmalz, H. G. (1998). *Angew. Chem. Int. Ed.* **37**, 911–914. 10.1002/(SICI)1521-3773(19980420)37:7<911::AID-ANIE911>3.0.CO;2-O 29711489

[bb4] Li, Z., Moser, W. H., Deng, R. & Sun, L. (2007). *J. Org. Chem.* **72**, 10254–10257. 10.1021/jo702109e 18004872

[bb5] Li, Z., Moser, W. H., Zhang, W., Hua, C. & Sun, L. (2008). *J. Organomet. Chem.* **693**, 361–367.

[bb6] Rigaku (2005). *CrystalClear* (Version 1.36) and *CrystalStructure* (Version 3.70). Rigaku Americas Corporation, The Woodlands, Texas, USA.

[bb7] Roberts, S. M., Santoro, M. G. & Sickle, E. S. (2002). *J. Chem. Soc. Perkin Trans. 1*, pp. 1735–1742.

[bb8] Sheldrick, G. M. (2008). *Acta Cryst.* A**64**, 112–122. 10.1107/S0108767307043930 18156677

[bb9] Shi, X., Gorin, D. J. & Toste, F. D. (2005). *J. Am. Chem. Soc.* **127**, 5802–5803. 10.1021/ja051689g 15839674

[bb10] Tanaka, K. & Fu, G. C. (2001). *J. Am. Chem. Soc.* **123**, 11492–11493. 10.1021/ja011907f 11707133

